# Evaluating the impact of Community-Based Sociotherapy on social dignity in post-genocide Rwanda: study protocol for a cluster randomized controlled trial

**DOI:** 10.1186/s13063-022-06994-3

**Published:** 2022-12-20

**Authors:** Stefan Jansen, Japhet Niyonsenga, Chantal Marie Ingabire, Angela Jansen, Emmanuel Nzabonimpa, Noella Ingabire, Jeannette Kangabe, Emmanuel Sarabwe, Annemiek Richters, Theoneste Rutayisire, Epaphrodite Nsabimana

**Affiliations:** 1 Mental Health & Behaviour Research Group, College of Medicine and Health Sciences, University of Rwanda, Kigali, Rwanda; 2 Department of Clinical Psychology, College of Medicine and Health Sciences, University of Rwanda, Kigali, Rwanda; 3 Community-Based Sociotherapy, Kigali, Rwanda; 4 Anglican Church Rwanda, Byumba Diocese, Byumba, Rwanda; 5 Prison Fellowship Rwanda, Kigali, Rwanda

**Keywords:** Community-Based Sociotherapy, Social Dignity Scale, Reconciliation, Psychosocial

## Abstract

**Background:**

Community-Based Sociotherapy (CBS) is an approach that was introduced in Rwanda in 2005, with the aim of improving psychosocial well-being among its participants and facilitating reconciliation processes. Over the years, CBS has been adapted contextually and the effectiveness of the approach has been measured in different ways, using qualitative and quantitative study designs. This study specifically assesses the effectiveness of CBS in terms of fostering the social dignity of participants as the primary outcome.

**Methods/design:**

A cluster randomized controlled trial design with person-level outcomes whereas the CBS treatment is delivered at the cluster level. A total of 1200 eligible participants will be randomly assigned to two groups in a 1:1 ratio. Participants in the intervention group will receive the CBS intervention, while the control group will be waitlisted. The primary outcome measure is a self-designed and psychometrically validated Social Dignity Scale. The secondary outcome measures will be the WHO (Five) Well-Being Index (WHO-5), the Multidimensional Scale of Perceived Social Support (MSPSS), the Posttraumatic Stress Disorder Checklist for DSM-5 (PCL-5), the Self-reporting Questionnaire (SRQ-20), and the perceived parental self-efficacy scale. The primary analysis will be performed following an intention to treat analysis, using generalized estimating equation modeling.

**Discussion:**

We expect this cluster randomized controlled trial to provide insight into the effectiveness of CBS on social dignity and secondary psychosocial outcomes among its group participants, who have different socio-historical backgrounds including genocide survivors, perpetrators, bystanders and their descendants, people in conflicts (family/community), and local leaders. This study will inform CBS implementers, policymakers, practitioners, and other stakeholders on the role of social dignity in interventions that focus on psychosocial healing.

**Trial registration:**

ISRCTN ISRCTN11199072. It was registered on 2 April 2022.

**Supplementary Information:**

The online version contains supplementary material available at 10.1186/s13063-022-06994-3.

## Introduction

The twentieth century was characterized by multiple genocidal events. This includes the genocide against the Jews, known as the Holocaust between 1941 and 1945; the Armenian genocide in 1915; the Cambodian genocide between 1975 and 1979; the genocide in the former Yugoslavia between 1992 and 1995; and the 1994 genocide against the Tutsi in Rwanda [1, 2]. Genocides leave long-lasting psychological and social harm to victims and perpetrators [3, 4]. During the 1994 genocide against the Tutsi, Rwanda was immersed in a brutal wave of organized violence in which approximately one million Rwandans lost their lives in a period of only 3 months [2, 5]. The 1994 genocide against the Tutsi in Rwanda, as opposed to other places where genocidal acts happened, has been massively executed by people known to the victims. By 1996, about 120,000 people were jailed due to genocide-related crimes (i.e., killings, rape, and destroying properties) [6], and later some of them returned to their community. After the release of prisoners, survivors had no other choice than to live alongside their perpetrators in the same neighborhood in post-genocide Rwanda.

A sizeable proportion of Rwandans were exposed to traumatic events during the genocide period resulting in a high prevalence of mental health problems [7]. Thirty-seven percent of men and 35% of women were exposed to at least one traumatic incident, such as being raped, witnessing an unnatural death, or being forced to escape their homes [7, 8]. Many Rwandans fled to neighboring countries and part of them came back a few years later [9, 10]. Consequently, mental disorders, including post-traumatic stress disorder (PTSD), depressive disorders, psychotic disorders, and substance use disorders, are the most often diagnosed disorders in the adult population in Rwanda today [11–15]. Worryingly, studies have shown that the transmission of trauma and PTSD, violence, and propensities to divisionism across generations is an issue of concern in the Rwandan community [16, 17]. In 1994, post-genocide, the entire health care infrastructure including the mental health care infrastructure was destroyed, while the needs were overwhelming. Efforts to rebuild the country led to a more stable situation. National (public and private) and international interventions addressing the negative consequences of genocide, such as trauma, impunity, a destroyed social fabric, and poverty, were implemented with notable positive gains.

The Community-Based Sociotherapy (CBS) intervention is one of the programs initiated in Rwanda to address the psychosocial needs of the Rwandan population in the aftermath of the 1994 genocide against the Tutsi and to restore the social fabric. The intervention is built on six consecutive phases, each focusing on a specific theme: safety, trust, care, respect, new life orientation, and memories. Throughout these phases, the following principles are applied: interest, equality, democracy, here and now, responsibility, participation, and learning by doing [18]. Compelling evidence shows that this psychosocial peacebuilding intervention promotes social cohesiveness, psychological well-being, reconciliation, and economic development for the study participants in Rwanda [8, 19]. CBS is also evidenced in other conflict/post-conflict settings, including in the Democratic Republic of Congo [20] and Liberia [8].

Furthermore, exploratory studies highlighted challenges related to the psychosocial reintegration of released prisoners. After their release, they usually go back to their respective families and communities, where local realities have changed. It is difficult for them to adapt to these new realities, especially when they still present feelings of worthlessness, self-stigma, and guilt. The studies mentioned above and the challenges still observed have informed the current “Mvura Nkuvure: Intergenerational healing and community reconciliation for sustainable peace” project that is being implemented by CBS Rwanda and its partners Anglican Church Byumba Diocese (EAR-D) and Prison Fellowship Rwanda (PFR) since November 2018 and Duhumurizanye Iwacu Rwanda (DIR) since December 2021.

Although CBS is a well-established intervention that can be adapted to and adopted by different contexts [8, 18], little is known about its effectiveness in increasing the social dignity of its participants. The choice for selecting “social dignity” as our primary outcome was made based on elaborate qualitative work with the CBS experts that resulted in identifying improved “social dignity” as the main outcome variable of their intervention. In a series of workshops, we explored the question of what CBS experts conceive to be the main indicators that together constitute the main outcome of the CBS intervention. We used structured equation modeling as the analytical framework to guide this exercise. This exercise led to the identification of a number of phases and principles (i.e., safety, trust, care, respect, equality, democracy) that can be understood in terms of long-term changes that CBS experts wish to see in participants after they have graduated from the intervention. Through an iterative process of literature review [21–24], interpretative work with CBS experts, and psychometric validation work on pilot data, we established an operational definition of “social dignity” within context and developed a validated questionnaire to measure the concept. We are preparing a separate publication on the contextualized conceptualization of social dignity and the development and validation of the social dignity scale [in preparation]. Improved mental health, higher levels of reconciliation, and increased social-economic wellbeing are understood as secondary outcomes to this primary outcome. We opted for a cluster design because of the nature of the CBS intervention. As CBS groups are composed of 10 to 15 people, these formed the natural clusters for our RCT study.

### Study objective

- ➣ To examine whether CBS enhances social dignity among its participants.
- ➣ To evaluate whether CBS improves mental health and psychosocial well-being among its participants.
- ➣ To investigate whether CBS’s impact on social dignity is mediated by improving mental health and psychosocial well-being.

### Study hypotheses

- ➣ CBS increases social dignity among its participants.
- ➣ CBS improves mental health and psychosocial well-being among its participants.
- ➣ CBS’s impact on social dignity is mediated by the improvement of mental health and psychosocial well-being.

### Trial design

This study will use a two-level cluster randomized controlled trial design with person-level outcomes and treatment delivered at the cluster level (the allocation fraction 1:1). One arm will serve as a control (control). At the same time, the other will receive the CBS treatment (Intervention). The design and report of this clinical trial protocol follow the Standard Protocol Items: Recommendations for Interventional Trials (SPIRIT) 2013 statement (Additional file 1), [25].

## Methods

### Setting and recruitment

This trial will be conducted from March 9, 2022, to March 2023 at different community centers of ten districts of Rwanda: Gasabo, Karongi, Rubavu, Rulindo, Burera, Gatsibo, Gicumbi, Nyanza, Muhanga, and Nyamagabe. The first endline survey will be done after the full cycle of fifteen weekly sociotherapy sessions whereas the second endline survey will be done 9 months after the baseline survey. The study will recruit participants through CBS facilitators based on the inclusion criteria of the trial. A total of 1200 participants who meet the eligibility criteria will voluntarily participate in the trial and will be asked to sign the informed consent form that has been approved by the ethics committee before enrolment. Participants from both rounds will be recruited in different (not neighboring) cells to minimize spillover.

### Eligibility criteria

#### Inclusion criteria

Everyone who is invited by a CBS facilitator for eventual participation is eligible. A CBS facilitator is a member of the community who has been selected and trained by CBS as an organization following criteria such as wisdom, integrity, openness, and capacity to care. The recruitment of participants in Sociotherapy is done following CBS guidelines on recruitment and involves CBS facilitators and sometimes community leaders. Basically, the recruitment is a community-based activity where CBS facilitators through word to mouth identify and visit potential participants of whom they become aware that they could potentially benefit from psychosocial support offered through CBS.

#### Exclusion criteria

Those who have gone through CBS in previous projects will not be eligible.

### Ethical issues

Ethical approval has been granted by the Institutional Review Board of the College of Medicine and Health Sciences at the University of Rwanda. The trial has been registered with ISRCTN (ISRCTN11199072, registered on April 2, 2022). Eligible participants will be informed about this trial and sign the consent forms before participating in the study. The trained data collectors will obtain informed consent from trial participants invited by the CBS facilitators. To comply with ethical considerations, participants assigned to a waiting list (control groups) will participate in the intervention after the final evaluation exercise (nine months later). All data collected for the purpose of this study will remain confidential and accessible only to the research team. All information will be stored in a password-protected computer.

### Randomization

University of Rwanda (UR) researchers, who are performing an external evaluation, will randomly select 80 groups from the CBS organization list of 137 groups. After baseline data collection from 80 groups, the computer-generated randomization sequence numbers will be generated using the SPSS (Version 28.0) statistical software. This indicates that 57 groups will be excluded from the study. The UR researchers will randomly assign the groups of participants (i.e., clusters) to either the intervention or control arms, observing a 1:1 ratio. The two rounds of endline data will be collected immediately after the 15-week intervention and 9 months post-intervention. At the baseline, the data collectors and study participants will be blinded. At endline, we will use the same blinding communication strategy. However, in reality, we do not expect the data collectors and study participants to be blinded anymore, as study participants become easily aware of which group they belong to, and they typically share this information with each other. In our experience implementing comparable RCT research, despite our efforts to create believable placebo intervention for the control group and despite dedicated efforts to blind data collectors and participants, we have observed that blinding in reality did not work.

### The intervention

#### Explanation for the choice of comparators

In this trial, the intervention groups will receive CBS whereas the control group will be on a waiting list to estimate the relative effect of CBS on social dignity and the secondary outcomes. To comply with ethical considerations, participants assigned to a waiting list (control groups) will also participate in the intervention after the evaluation exercise (six months later).

#### The intervention of usual care (control)

In this study, the usual care is access to public health and other treatment options that are available in the community. The CBS intervention is an intervention that takes place in Rwanda. They select people from the community (as described above) and assist them through the CBS intervention. Our research design aims to test the ecologically valid impact of the CBS intervention, meaning that we compare people who received the intervention versus people who are eligible for intervention (and were put on the waiting list). We opted for this approach as we could not create a meaningful intervention that could be used in the control group that would not generate a positive intervention effect (that would change the measures of our outcome variables). In addition, for ethical reasons, we did not want to engage the control group participants in placebo intervention activities that would not create a meaningful improvement in their lives.

#### Intervention description

The Community-Based Sociotherapy (CBS) intervention is one of the programs initiated in Rwanda to address the psychosocial needs of the Rwandan population in the aftermath of the 1994 genocide against the Tutsi and to restore the social fabric. CBS is a psychosocial peacebuilding intervention that has been shown to promote social cohesiveness, psychological well-being, reconciliation, and economic development among the populations of Rwanda [8, 19]. CBS is also evidenced in other conflict/post-conflict settings, including in the Democratic Republic of Congo [20] and Liberia [8].

**Fig. 1:**
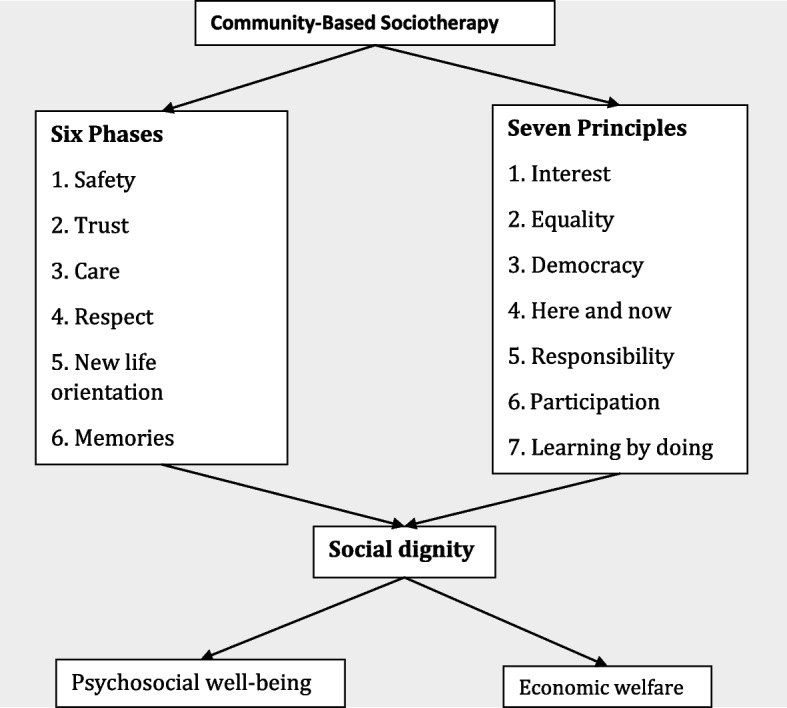
Conceptual framework

In CBS, a group of 10 to 15 people gather in a circle to discuss their daily life problems and psychosocial distress, which turn out to often be related to their past and how it has affected their psychosocial health. The intervention is built on six consecutive phases, each focusing on a specific theme: safety, trust, care, respect, new life orientation, and memories. Throughout these phases, the following principles are applied: interest, equality, democracy, here and now, responsibility, active participation, and learning by doing [18] (see Fig. 1). In the sessions, the sociotherapy facilitators include exercises and cultural games and expressions, which contribute to cohesive group dynamics.

The discussion of the psychosocial difficulties impacting both people and their community unfolds in particular over the course of the first four intervention phases, while the future life orientation and learning how to manage painful memories is the main focus of the final two intervention phases. The order in which safety, trust, care, and respect are established in the sociotherapy groups enables people to suspend the discussion of tough topics until an emotionally safe group environment has been created. Processing negative emotions takes place throughout the phases while in the sixth and final phase people are invited to focus on dealing with negative emotions in a constructive way as part of a new life orientation formation [8, 18, 26].

Every phase of sociotherapy is intended to advance the psychosocial health of the participants and their propensity to peaceful attitudes. The different phases help participants to not view life only in negative ways, but also to imagine and learn about establishing positive life experiences. The first phase, “safety”, provides a setting where each participant feels secure during group talks. In the second phase, “trust”, participants join together to rebuild the trust toward people and institutions lost by participants (perpetrators and survivors) following a history of trauma. Bringing social cohesiveness and psychological healing to each group member depends on re-establishing trust between cognitive and social-emotional attention and care for the individual inside the group [8, 26]. The third phase, “care”, is centered on people who had encountered bad experiences and had not been taken care of, had lost their ability to take care of themselves, or found it challenging to take care of others. In this phase, each participant grows sympathetic toward certain group members, the group dynamics come to life, and the group now serves as a conduit for social events [27]. In the fourth phase of “respect”, participants discuss how they were not respected and were prevented from respecting others due to various causes that negatively impacted their psychosocial well-being. Respect must be rebuilt inside the group, and each group member goes through the process of building self-confidence and fostering one’s dignity in relation to that of others [26]. In the fifth phase, “new life orientation,” participants compare their behaviors, feelings, and emotions before group sessions, as well as the benefits of group participation and their renewed emphasis on improving their psychosocial health. In the sixth and final phase, “memories,” individuals recall past traumas and relive the emotions that broke their lives’ equilibrium and relations with others. Participants recreate new meanings of their experiences after recalling both positive and negative situations [26, 27].

Since the start of the intervention of CBS, the implementation has gone hand in hand with monitoring, evaluation, and research activities for effective and evidence-based planning. Studies conducted on the CBS intervention demonstrated the effectiveness of the intervention in contributing to improved mental well-being, social connections, healing and reconciliation, social capital, reduction of partner violence, active civic participation, improved economic development, and peacebuilding [18, 24]. It also increased levels of trust among people, who subsequently start grasping the benefits of social networks (social capital), like expanding skills, accessing information, and joining efforts for their development either at the family level (bonding) or the broader community level (bonding and bridging), [28, 29].

#### Criteria for discontinuing or modifying allocated interventions

Over the past 15 years that the CBS intervention has been used and researched, both through qualitative and quantitative work, we have amassed good evidence that the intervention does not cause harm. On an individual level, the trial participants assigned to the study intervention will be discontinued whenever the participants (1) request to withdraw from the study or (2) do not comply with the group rules and regulations during the first session (including secret keeping).

#### Strategies to improve adherence to interventions

Individual face-to-face follow up by CBS facilitators with participants encouraging them to adhere to the group sessions, and further encouragement during the group sessions are part of the normal CBS practice and will also be implemented during the study.

#### Relevant concomitant care permitted or prohibited during the trial

To the best of our knowledge, there is no concomitant intervention implemented in the catchment area of the Community-Based Sociotherapy. The participants will be allowed to join any intervention that may be created during the trial period without the need to ask for the permission of the CBS facilitators. To control for co-intervention bias, we will monitor whether individuals are taking part in similar group therapeutic interventions [30].

#### Provisions for post-trial care

There is no risk associated with participating in this study, except perhaps the risk of some discomfort that participants may feel as we ask questions about their personal life. Moreover, a team of clinical psychologists will provide psychological support in the intervention of emotional crises. No direct compensation will be allocated to participants. Participants in the control group are waitlisted and will be invited to join CBS groups in future post-study intervention cycles.

### Outcomes

#### Primary outcome measure

Following extensive qualitative work with the CBS experts, we defined the impact of the CBS intervention in an itemized way. After we searched the literature for concepts that relate to the lists of items and through iterative feedback working loops between CBS experts and the literature, we came up with a contextually developed definition of social dignity, which CBS experts recognized as an ecologically valid primary outcome measure for CBS.

All items of the self-designed social dignity scale will be summed up to form a total score that will be used to estimate the primary outcome before and after the CBS intervention (baseline, endline1, and endline2). Each item is scored on a 5-point Likert Scale ranging from 1 (never) to 5 (always). We believe that this sum will give a valid measure of social dignity, as qualitative work preceding the trial did not indicate that we should weigh any item of the questionnaire higher than the other.

#### Secondary outcome measures

Measured before and after the CBS intervention (baseline, endline1, and endline 2):

1. Sociodemographic characteristics (age, sex, marital status, location, education, occupation, and social category) will be measured using a sociodemographic questionnaire.
2. Personal well-being will be measured using the WHO (Five) Well-Being Index [31].
3. Perceived social support will be measured using the Multidimensional Scale of Perceived Social Support [32]
4. PTSD symptoms will be measured using the Posttraumatic Stress Disorder Checklist for DSM-5 with LEC for PCL-5 [33].
5. Depression symptoms will be measured using the Patient Health Questionnaire (PHQ-9), [34].
6. Attributed dignity will be measured using the Jacelon Attributed Dignity Scale [35].
7. Perceived parental self-efficacy will be measured by the perceived parental self-efficacy scale [36].

### Data collection timeline

**Fig. 2:**
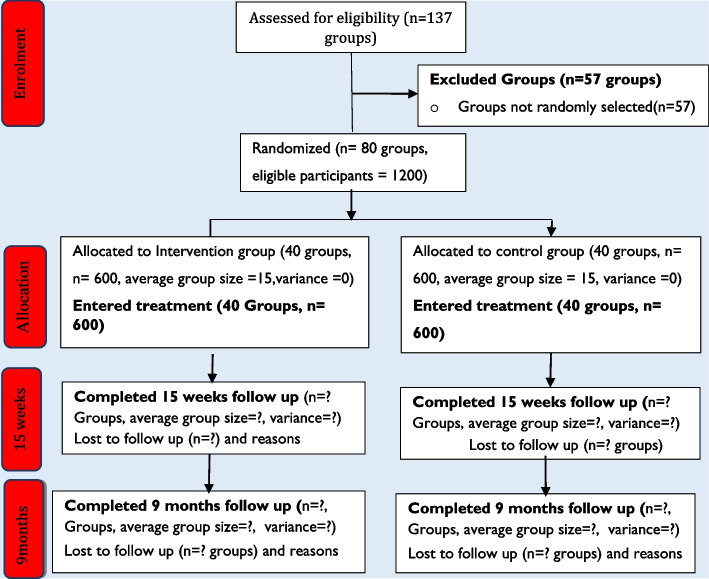
The schematic chart

The schematic chart and study schedule are shown in Fig. 2 and Table 1.

**Table 1:**
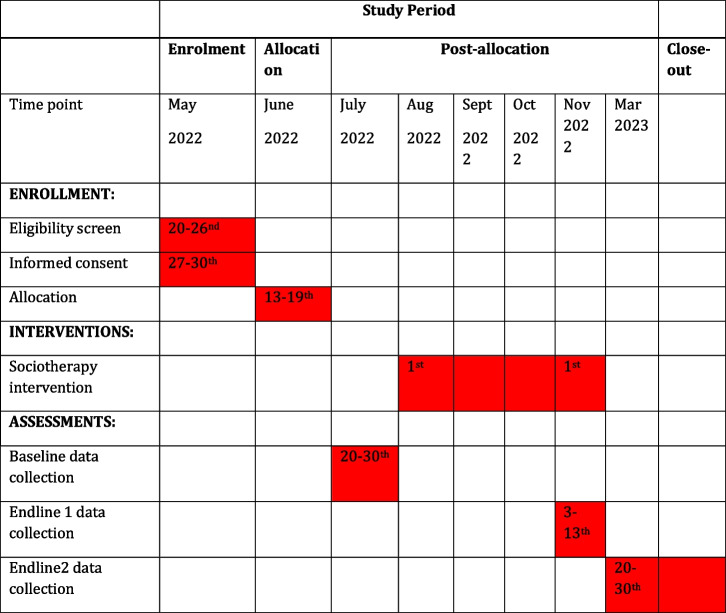
Study schedule

Note: shaded cells indicate the main activity and their corresponding study dates

### Sample size

The sample size was estimated based on pilot study results. As there was no similar previous study allowing to asses intraclass correlation, the authors of this study have conducted a pilot study on a sample of 559 people. The objectives of the pilot study were (1) to test the psychometric properties of the Social Dignity Scale and (2) to assess potential Intra-CBS groups correlation. The results from the pilot study revealed an intraclass correlation of 0.15. The targeted treatment effects were established in terms of clinical judgment, not through the pilot of data [37]. According to previous CBS experience, an average of 3 out of 15 participants in a CBS group dropout the intervention without achieving at least 11/15 required sessions. All enrolled CBS groups are facilitated to the end of the intervention. Considering an intraclass correlation coefficient, Rho = 0.15 and a standard Alpha = 0.05, an a priori power calculation using the Optimal Design Software [38] revealed that 80 CBS groups (40 control and 40 intervention) composed of 12 participants each group would make it possible to achieve the standard power of 0.80 while detecting a small Cohen’s *d* effect size of 0.3. The required total sample was then found to be 960 (i.e., 12 × 80) participants. However, assuming a minimalistic response rate of 96% according to a previous similar social study in Rwanda evidencing that it ranges from 96% [39] to 99.8% [40], we would need to recruit 1000 participants. To provide a buffer against potential attrition-related problems, at least 15 recruited beneficiaries of CBS that fulfil the inclusion criteria will be included in the study making the final sample size to recruit up to 1200 participants.

### Assignment of interventions: allocation

#### Sequence generation

The computer-generated randomization sequence numbers will be generated using the SPSS (Version 28.0) statistical software. Details of the procedure are described in the “Randomization” section.

#### Concealment mechanism

Participants will be randomized using the computer-generated randomization sequence numbers by the University of Rwanda Researchers. Allocation concealment will be ensured, as the researchers will not release the randomization code before the participants provide the consent forms to avoid the influence of knowledge of the group to which they will be allocated if they join the trial [41].

### Assignment of interventions: blinding

A detailed description of the blinding procedures is described in the “Randomization” section.

### Data collection and management

#### Plans for assessment and collection of outcomes

A team of 20 data collectors will be recruited and given a 3-day training on evaluation objectives, introduction to ethical considerations, the content of the questionnaire, and electronic data capture followed by practical sessions to ensure accurate, reliable, and consistent data collection. In addition to self-designed measures on social dignity and reconciliation, we will use standardized questionnaires on mental health and psychosocial wellbeing. The interviews will take about 45 min.

### Plans to promote participant retention and complete follow-up

No additional strategies will take place to improve retention and completion by participants in this trial, in keeping with the ecological design of this study.

### Data management

Data will be collected in an electronic data management system (Kobotoolbox) and extracted for statistical analysis. One research assistant will be responsible for data management. He will check the data quality during and after data collection in addition to cleaning the data. Additionally, session attendance will be weekly recorded by the CBS facilitators. All information will be stored in a password-protected computer.

### Confidentiality

Data will be collected in an electronic data management system (Kobotoolbox) and extracted for statistical analysis. A file containing all evaluation-related documents (attendance lists, ID logs, informed consent forms, etc.) will be kept in a secure location at the field for the entire duration of the project. In short, data collected for this study will remain confidential and accessible only to the research team. All information will be stored in a password-protected computer.

### Data collection

A team of 20 data collectors will be recruited and given a 3-day training on evaluation objectives, the content of the questionnaire, and electronic data capture, followed by practical sessions to ensure accurate, reliable, and consistent data collection. Quantitative data will be collected in an electronic data management system (Kobotoolbox) and extracted for statistical analysis. A file containing all evaluation-related documents (attendance lists, ID logs, informed consent forms, etc.) will be kept in a secure location at the field for the entire duration of the project. In addition to standardized questionnaires on mental health and psychosocial well-being, several questions related to everyday life like trust, care, respect, safety, responsibility, perceived social support, traumatic events, PTSD, and attributed dignity will be asked. The interviews will take about 45 min.

### Statistical methods

#### Baseline characteristics

Baseline characteristics, both at the cluster (i.e., group size, group location) and individual levels (age, sex, marital status, location, education, occupation and social category), will be cross-tabulated according to the allocated group to check for appropriate balance and to provide an overview of the study population. The baseline characteristics of each group will be summarized as the mean, standard deviation, and range for continuous, approximately normally distributed variables; medians, interquartile range, and range for continuous, skewed variables; and frequencies and percentages of individuals in each category for categorical variables. The formal statistical comparison at the baseline of randomized groups will not be undertaken.

#### Primary analysis

The primary analysis will be performed following an intention-to-treat analysis. Primary outcome measures will be the post-intervention continuous total score of the social dignity questionnaire and its subscales based on socio-therapy phases. As the researchers are interested in discussing change and the correlates of change [42], the primary analysis will compare intervention vs control groups on their mean change in social dignity between baseline and endline, and between baseline and endline2 separately. To counter the problems associated with their use, the baseline scores will be incorporated into the model as a control [42, 43]. The mean change scores in social dignity will be incorporated into the GEE model as a dependent outcome. GEE will help to adequately account for intra-cluster correlation by incorporating in the model the random CBS groups/cluster-specific effect. Our primary interest is at the individual level and the CBS groups/cluster level will be regarded as a nuisance that must be considered for valid inferences [44].

Allocated groups (control versus intervention) will be regarded as fixed effects while clusters and time points are regarded as random effects in the model. With this approach, we will be able to separately model the mean response at the individual level and the cluster within-cluster association. The model will be adjusted using pre-defined covariates. Our primary interest is at the individual level, and the cluster level is regarded as a nuisance that must be considered for valid inference. We will test the robustness of the results by repeating the analyses using mixed random effects regression analysis to compare the two groups and a standard *t*-test to compare the means of the outcomes at the individual level [45, 46]. The estimated difference in mean change from baseline to endline and the corresponding 95% confidence interval (CI) will be presented. Secondary outcomes will be analyzed using the same method as the primary outcome.

Notably, no formal interim analysis will be conducted on the primary and secondary outcomes because we do not have the design issues, anticipated harm, or early intervention effects for the respondent which are the most cited reasons for interim testing [12, 47]. A pool of previous qualitative studies has proven that CBS is a well-established intervention with a well-predefined number of required sessions [19]. As such, no risk or inconvenience was found to be associated with CBS. The participation rate will be captured as usual intervention monitoring exercise to estimate its covariation effects. However, data monitoring will be done by UR team to ensure that the data collected are in the right format at baseline and both end-lines.

#### Subgroup analysis

Exploratory subgroup analyses of the following possible interactions will be undertaken to assess whether the effect of sociotherapy intervention is modified by the prespecified covariates: survivors, perpetrators, and youth. These subgroup analyses will be performed by adding the interaction term between the allocated group and the subgroup variable into the generalized effect model. Given the limited power to detect treatment–subgroup interactions, analyses of the results will be interpreted with caution. All subgroups will be analyzed using the intention to treat the population. Wherever applicable, to compensate for multiple comparisons, this study will employ a Bonferroni correction factor.

#### Interim analyses

No formal interim analysis will be conducted on the primary and secondary outcomes. However, data monitoring will be done to ensure that the data collected are in the right format at baseline and at both endlines. This will be conducted by UR researchers, who are performing an external evaluation.

## Methods in analysis to handle protocol non-adherence and any statistical methods to handle missing data

The reasons for missing data will be reported and if missing data is < 5%, multiple imputation will be used to handle missing data [48] using SPSS (version 28).

## Plans to give access to the full protocol, participant-level data, and statistical code

There are no plans for granting public access to the full protocol, participant-level dataset, and statistical code beyond the publication of this protocol.

### Adverse event reporting and harms

There is no known adverse event or harm associated with participating in this study. Nevertheless, given that this study is being carried out in a post-genocide context, we could expect participants to experience emotional distress. Therefore, any adverse events that might arise during the conduct of this trial will be duly recorded by data collectors, who will thereafter report to field leaders. This will lead to immediate follow-up by a team of clinical psychologists who are on site and will provide psychological support when needed.

### Plans for communicating important protocol amendments to relevant parties

All protocol amendments will be reviewed and approved by the Institution Review Board of the College of Medicine and Health Sciences for prior approval or notification. The Principal Investigator will sign and date the approved protocol amendment before implementation. Any departures from the protocol will be documented in the participant file.

#### Dissemination plans

Planned publications in high-impact peer-reviewed journals. We will also disseminate locally at community and policy levels. And we provide detailed feedback to CBS. Participant-level dataset and statistical codes will be made available on reasonable request from the principal investigator.

#### Trial status

This is the original version of the protocol, issued in April 2022. The recruitment phase is planned to start in July 2022 and end approximately by August 2022. Any changes or protocol amendments will be accounted for in the public study record available on clinical trials. gov with ID: ISRCTN11199072.

## Discussion

This is the first study that evaluates the effectiveness of CBS on social dignity among its participants, including genocide perpetrators, survivors, their descendants, people in conflicts (family/community), and local leaders. This represents an ecologically valid approach as the primary outcome variable is the result of qualitative work with CBS experts which lead to the conclusion that this outcome best describes the true impact CBS intends to have (and drives secondary outcomes). Compelling evidence shows that CBS contributes to improved mental well-being, social connections, restorative justice, healing and reconciliation, increased trust and social capital, reduction of partner violence, active civic participation, improved economic development, and peacebuilding [28, 29, 49]. The proposed study will allow us to obtain a deeper understanding of the effectiveness of CBS on a psychometrically validated measure that reflects the functioning of CBS as understood by CBS experts, and will help us understand how this impact relates to secondary which are well-established and theoretically founded. Concretely, this study will allow us to measure the effectiveness of CBS among individuals dealing with the consequences of the 1994 genocide against the Tutsi on social dignity and related secondary outcomes. We hypothesize that participants in the intervention group will show improved social dignity and secondary outcomes compared to those in the control groups.

More broadly, this study could inform CBS implementers, policymakers, and other stakeholders and practitioners on the role of social dignity in interventions that focus on psychosocial healing interventions.

## Supplementary Information

**Additional file 1. **SPIRIT checklist.

## Data Availability

The final data set for the proposed study will be available upon reasonable request from the corresponding author.
